# Synthesis and insecticide evaluation of some new oxopropylthiourea compounds as insect growth regulators against the cotton leafworm, *Spodoptera littoralis*

**DOI:** 10.1038/s41598-023-39868-y

**Published:** 2023-08-11

**Authors:** Ahmed M. El-Saghier, Laila Abosella, Gamal A. Aborahma, Esmail O. Elakesh, Antar A. Abdelhamid, Mohamed A. Gad

**Affiliations:** 1 Chemistry Department, Faculty of Science, Sohag University, Sohag, 282524 Egypt; 2 Medicinal Chemistry Department, Faculty of Pharmacy, Sabratha University, Sabratha, Libya; 3 Medicinal Chemistry Department, Faculty of Pharmacy, Minia University, Minia, Egypt; 4 Chemistry Department, Faculty of Science, Al Zawiya University, Al Zawiya, Libya; 5 Chemistry Department, Faculty of Science, Al-Baha University, 1988 Al-Baha, Saudi Arabia; 6 Agriculture Research Center, Research Institute of Plant Protection, Giza, 12112 Egypt

**Keywords:** Chemical biology, Evolution, Structural biology, Chemistry

## Abstract

In this project we aim to share in increasing the production of the most important non-food agricultural product i.e. cotton via protection of it is plant. The usage of safe alternatives to the pesticides has become crucial due to several serious issues associated with the use of insecticides. Therefore, the families of new eco-friendly organic compounds that contain manly oxopropylthiourea scaffold will synthesis in their pure state by using green procedures. This compounds includes (i) poly functional substituted oxopropylthiourea, (ii) dihydroquinoline carboxylic acid, In second category, the structure of this compounds which may be related to the most famous insect growth regulators insecticides, will confirmed by elemental and modern spectroscopic analyses (such as IR, UV, ^1^HNMR and ^13^CNMR). In the final category, the synthesized compounds was checked toward the second & forth instar larvae of cotton leafworm, *Spodoptera littoralis*. The present data proved that values of LC_50_ of the most effected synthesized compound **8** was 2.412 ppm in which LC_50_ for commercial lufenuron was 2.295 ppm. Component **8** may be particularly effective due to the presence of fluorophenyl, cyanoacetamide, and carboxalic acid groups in their chemical makeup. In an additional effort to slightly improve insecticidal compounds, evaluation of the latent effects of the examined components on a number of biological parameters, such as adult longevity, pupal weight, proportion of normal, deformed pupae, & adult emergency, fecundity, & egg hatchability, was carried out.

## Introduction

**Figure 1:**
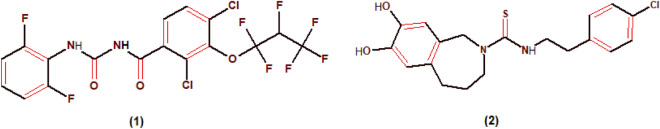
Chemical structure of Lufenuron (**1**) and Capsazepine (**2**).

Insect pests compete fiercely with people for agricultural resources because they harm and decrease the productivity of most crops^1^. Vegetative shoots are chewed, stems are eaten, or roots or tubers are consumed by biting insects including locusts, beetles, and Lepidoptera larvae^2,3^. The moth species *Spodoptera littoralis* (Boisduval, 1833) belongs to the Noctuidae family and is widely distributed throughout Africa, Mediterranean Europe, and the Middle East^4^. For many nations, it is well known that the cotton leaf worm causes significant financial losses^5^. Cotton, potatoes, maize and vegetables are just a few of the highly harmful polyphosphorous plant species that the highly venomous *S. littoralis* moth feeds on^6^. It also consumes more than 100 other species. The largest problem facing pesticide research now is likely pesticide resistance. In order to create an effective control strategy for *S. littoralis* in the future, it is necessary to evaluate various insecticides from various chemical groups with various modes of action as well as some of their combinations^7^. In an effort to boost the effectiveness of insecticides against *S. littoralis* while decreasing the amount of pesticides released into the environment, which is important from the perspective of environmental safety, the combination of such bioactive compounds with insecticides was researched^8–10^. In particular, when it comes to pesticides, the majority of urea compounds have a wide range of bioactivities, including insecticidal^11^, antifungal^12^, herbicidal^13^, and antitumor^14^ effects. In order to improve the insecticidal profile of triazone insecticides and mimic the molecular mechanism of action of pymetrozine and other TRP antagonists, thiourea bridge groups were added. This led to the synthesis of four different novel triazone analogues, which were then studied. For instance the insect growth regulator lufenuron (match), which has a wide range of bioactivity against lepidopteran and coleopteran pests, suppresses the development of chitin, throws off the hormonal balance during the moulting process, and does both. (Fig. 1)^15^.

In this investigation, we examine how these synthetic IGR-related chemicals affect *S. littoralis*. This study compares the toxicities of these synthesized compounds and the standard lufenuron against *S. littoralis*^16,17^. accordingly, this work was aiming to (1) designing & characterizing of different compounds of poly functional substituted oxopropylthiourea. (2) Investigating their insecticidal effectiveness toward *S. littoralis*. Our data is measured the first phase in insecticide discovery which it might be appreciated for insecticidal activity companies to enhance novel insecticides toward noctuid moths. These new compounds' insecticidal properties were assessed, and structure–activity connections were examined.

## Materials and methods

### Chemical compounds and reagents

All synthesized compounds were estimate melting point by a Fisher-John mechanical technique. In our search the instrumentations, chemical compounds & solvents have been acquired from Sigma-Aldrich. The infra-red spectra of the designed products were analyzed employed potassium bromide technique, ^1^HNMR & ^13^CNMR were recorded on spectrometer model Bruker Advance 400 MHz. A reference Lufenuron insecticides were acquired from Sigma-Aldrich.

### Laboratory bioassay screening

All synthetic oxopropylthiourea derivatives were tested for their insecticidal bioactivity using the industry-standard leaf dip bioassay techniques.^18–23^ preparation of the compound stocks to create 1000 ppm, 0.1 g of compounds 1–10 were dissolved in 5 mL of Dimethyl formamide & combined with 100 mL of distilled H_2_O. Until usage, the stocks were kept in a refrigerator. The target substances' test results were noted, & the concentrations needed to destroy 50% (LC_50_) of *S. littoralis* larvae were calculated. Oxopropylthiourea derivatives were employed in five different concentrations, & 0.1% Tween 80 was employed as a surfactant. Castor bean leaf discs (nine centimeters in diameter) were dipped in the concentration under test for ten seconds, then fed to 2th & 4th larvae, which were roughly the same size and housed in glass jars (five lb). Each action carried out 3 times with ten larvae each. The castor bean leaf has been used in our study, they were collected from the Shandaweel research station geographic area, Sohag governorate, Egypt and we are confirmed that, it is accordance with relevant institutional, national and international guidelines and legislation.

### Statistical analysis

The mortality equalized via Abbott's formula^24^. Calculations of mortality setback line were measurably rummage via probity analysis^25^. Harmfulness index was strongminded via sun equations^26^. The mortality results of larval insect were estimated through employing probit analysis through a statistics (LDP-line) equation which estimate the LC_50_ values with 95% fiducially limits of lower, upper confidence limit and slope.

## Result & discussion

### Synthesis

**Figure 2:**
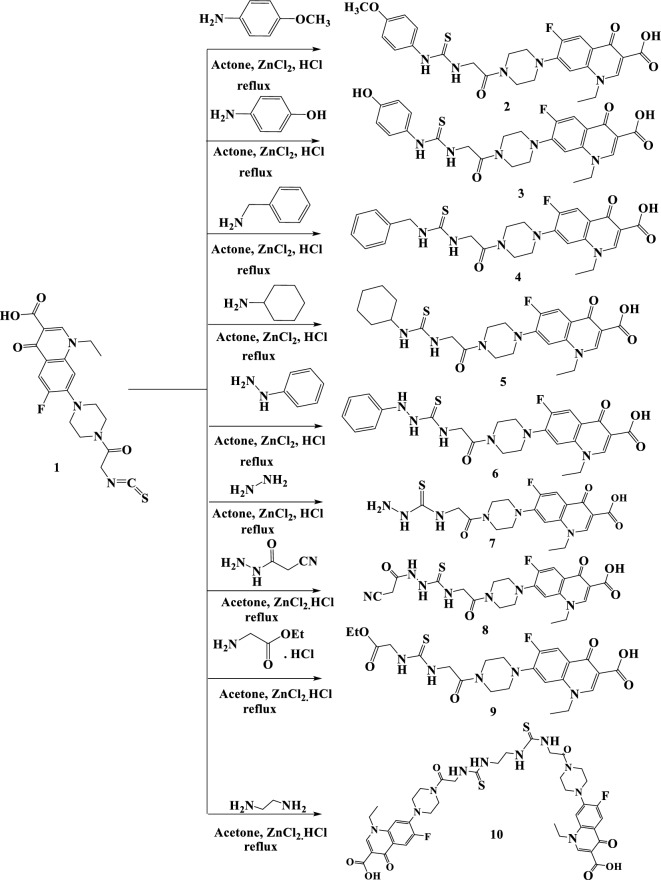
Designing of novel oxopropylthiourea derivatives (**2–10**).

The reaction of secondary amine 1-ethyl-6-fluoro-4-oxo-7-(piperazin-1-yl)-1,4-dihydroquinoline-3-carboxylic acid with chloroacetyl chloride in 1,4 dioxane to give 7-[4-(chloroacetyl)piperazin-1-yl]-1-ethyl-6-fluoro-4-oxo-1,4-dihydroquinoline-3-carboxylic acid, which reacted with ammonium thiocyanate, the vital intermediate 6-fluoro-7-[4-(isothiocyanatocarbonyl)piperazin-1-yl]-4-oxo-1,4-dihydro- quinoline-3-carboxylic acid (**1**) was synthesized^27–30^. Herein, the aimed synthesized products, which named as: 1-Ethyl-6-fluoro-7-(4-(((4-methoxyphenyl)carbamothioyl)glycyl) piperazin-1-yl)-4-oxo-1,4-dihydroquinoline-3-carboxylic acid **2,** 1-Ethyl-6-fluoro-7-(4-(((4-hydroxyphenyl)carbamothioyl)glycyl)piperazin-1-yl)-4-oxo-1,4-dihydroquinoline-3-carboxylic acid **3,** 7-(4-((Benzylcarbamothioyl) glycyl)piperazin-1-yl)-1-ethyl-6-fluoro-4-oxo-1,4-dihydroquino-line-3-carboxylic acid **4,** 7-(4-((Cyclohexylcarba- mothioyl)glycyl)piperazin-1-yl)-1-ethyl-6-fluoro-4-oxo-1,4-dihydroquino-line-3-carboxylic acid **5,** 1-Ethyl-6-fluoro-4-oxo-7-(4-((2-phenylhydrazine-1-carbonothioyl) glycyl)piperazin-1-yl)-1,4-dihydroquinoline-3-carboxylic acid **6,** 1-Ethyl-6-fluoro-7-(4-((hydrazinecarbonothioyl)glycyl) piperazin-1-yl)-4-oxo-1,4-dihydroquino-line-3-carboxylic acid** 7, 7**-(4-((2-(2-Cyanoacetyl) hydrazine-1-carbonothioyl)glycyl)piperazin-1-yl)-1-ethyl-6-fluoro-4-oxo-1,4-dihydroquinoline-3-carboxylic acid** 8,** 7-(4-(((2-Ethoxy-2-oxoethyl)carbamothioyl) glycyl)piperazin-1-yl)-1-ethyl-6-fluoro-4-oxo-1,4-dihydroquinoline-3-carboxylic acid** 9** and 7-(4-(((2-Aminoethyl)carbamothioyl)glycyl)piperazin-1-yl)-1-ethyl-6-fluoro-4-oxo-1,4-dihydroqui noline-3-carboxylic acid **10** were successfully prepared Fig. 2, the obtained yield is 50−70% through the following steps.

A solution of component **1** (4 mol) in dry acetone (20 ml), (4 mol) of amine derivatives, (1 mL) conc. HCl and (0.2 gm) anhydrous ZnCl_2_. The reaction mixture allowed to reflux about 8 h. Cooled and poured to H_2_O. The crystal product was collection via filtration.

### Toxicological effectiveness checking for 2nd larvae

The procedure was carried out in accordance with the published method as part of our ongoing research into bioactive thioura derivatives.^30,31^ According to Table 1 the result of synthesized target compounds **1–10** were tested against 2nd larvae insect of *S. littoralis*. The bioefficacy results of tested compounds against the 2nd larvae exhibit from high to low toxicological activity which LC_50_ values vary from 2.412 to 11.40 ppm in which the LC_50_ value of a reference lufenuron was 2.295 ppm. Moreover, the LC_50_ value of compounds **1–10** were 10.738, 3.810, 3.505, 5.943, 11.40, 4.648, 10.922, 2.412, 10.84 and 40.98 ppm respectively, in which lufenuron stander insecticide rate was 2.295 ppm. Consequently, the toxicity of designing product **8** toward second larvae instar insect of *Spodoptera littoralis* was nearly closed in insecticidal bioactivity than reference lufenuron. These results agree with Bhongade, et al., 2016 who referred that thiourea derivatives exhibited the highest toxic biological activity^32^.

**Table 1:** Insecticidal effectiveness of components **1–10** & Lufenuron as reference insecticide toward the 2nd & 4th larvae instar of *S. littoralis* after 3 days of treatment.

2nd instar larvae				4th instar larvae		
Comp	LC_50_ (ppm)	Slope	Toxic ratio^a^	LC_50_ (ppm)	slope	Toxic ratio
1	10.73	0.951 ± 0.259	0.213	15.10	0.816 ± 0.250	0.601
2	3.810	0.807 ± 0.271	0.602	12.78	0.793 ± 0.251	0.710
3	3.505	0.714 ± 0.265	0.654	10.18	0.676 ± 0.250	0.891
4	5.943	0.943 ± 0.250	0.386	14.66	0.850 ± 0.252	0.619
5	11.40	0.909 ± 0.256	0.201	17.68	0.968 ± 0.254	0.513
6	4.648	0.753 ± 0.263	0.493	13.10	0.759 ± 0.250	0.692
7	10.92	0.913 ± 0.257	0.210	16.84	0.715 ± 0.218	0.539
8	2.412	0.681 ± 0.270	0.951	9.531	0.977 ± 0.262	0.952
9	10.84	0.981 ± 0.262	0.211	16.54	0.907 ± 0.253	0.548
10	10.98	0.919 ± 0.255	0.209	16.77	0.939 ± 0.253	0.541
Lufenuron	2.295	0.688 ± 0.272	1	9.079	0.681 ± 0.251	1

Notes: Toxicity Ratio is calculated as lufenuron’s LC_50_ value for baseline toxicity/the compounds’ LC_50_ value.

### Toxicological effectiveness checking for adults 4th larvae

After 72 h of treatment, the objective products **1–10** exhibit varying degrees of insecticidal efficacy. Their respective LC_50_ values for the fourth larvae of *S. littoralis* were 15.10, 12.78, 10.18, 14.66, 17.68, 13.10, 16.84, 9.531, 16.54, and 16.77 ppm. According of this word, the toxicity of products **2** & **8** toward forth larvae of *S. littoralis* was nearly lufenuron after seventy two h in which LC_50_ value of compounds **8** and** 3** was 9.531 and 10.18 ppm and lufenuron was 9.079 ppm. On considering the toxicity line and slope we observed that the slope increase in the following order **8** > **3** > **2** > **6** > **4** > **1** > **9** > **7** > **10** > **5,** this order revealed that the homologous response of the treated strain of *S. littoralis* which presented variation in response against of target synthesized products.

### Biological studies

According the reported method to determinates the biological characteristics of the thiourea derivatives such larval and pupal duration, pupal weight, the percentage of normal, malformed pupae, and adult emergency, as well as the percentage of fecundity and egg hatchability, were assessed for the latent effects of the investigated component^32^. Activity of the synthetic target components **2, 3, 4, 6** and **8** under test on some biological characteristics of *S. littoralis*. Recently molted fourth instar larvae of *S. littoralis* were fed caster bean leaves treated with LC_25_ concentrations of the most poisonous Oxopropylthiourea derivatives **2, 3, 4, 6** and **8** for 48 h before being switched to untreated leaves until pupation as part of an investigation into the biological characteristics of the species. Tables 2 and 3 present the findings after recording the key biological characteristics.

**Table 2:** The biological characteristics of a laboratory strain of fourth-instar larvae of S*. littoralis* (Boisd.) were affected by the very poisonous, recently synthesized compounds **2, 3, 4, 6** and **8** at their LC_25_ values.

Tested compound	LC25 mg/L	Larval duration Days ± SE	Pupal duration Days ± SE	Weight (mg) ± SE	Normal pupae % ± SE	Deformed pupae % ± SE	Adult emergence % ± SE
8	1.75	18.61^a^ ± 0.20	11.32f. ± 0.20	263.72^e^ ± 0.22	36.56^e^ ± 0.56	16.23^a^ ± 0.34	73.35^c^ ± 0.51
3	2.03	16.35^b^ ± 0.20	13.65^e^ ± 0.20	272.14^d^ ± 0.19	72.51^d^ ± 0.40	15.62^b^ ± 0.33	65.23^d^ ± 0.86
2	2.35	14.56^c^ ± 0.01	14.20^d^ ± 0.01	281.60^c^ ± 0.16	81.25^c^ ± 0.35	7.65^c^ ± 0.30	68.31^b^ ± 0.57
6	2.56	11.95^d^ ± 0.20	15.33^c^ ± 0.20	286.15^b^ ± 0.14	90.25^b^ ± 0.80	5.65^d^ ± 0.20	80.24^b^ ± 0.38
4	3.65	10.25f. ± 0.20	16.85^b^ ± 0.20	395.14^a^ ± 0.11	94.68^a^ ± 0.25	3.23^e^ ± 0.17	89.61^a^ ± 0.61
Control		9.55^ g^ ± 0.20	17.33^a^ ± 0.20	298.0^a^ ± 0.29	95.21^a^ ± 0.29	3.20^e^ ± 0.17	93.41^a^ ± 0.62
LSD = 0.05		0.73	0.73	0.73	1.02	0.86	1.96

Letters mean the noteworthy differences between treatments in line with Duncan’s check SE = Standard error.

**Table 3:** The influences of the greatly toxic novelty designing components **2**, **3**, **4**, **6** and **8** at their LC_25_ values on the fecundity, fertility & adult longevity for *S. littoralis* (Boisd.) laboratory strain larvae that survived the fourth instar.

Tested compound	No. of eggs/female ± SE	Fecundity% ± SE	Egg hatchability% ± SE
8	702.39f. ± 14.58	22.85f. ± 0.20	42.36f. ± 0.20
3	956.33^e^ ± 10.25	42.65^e^ ± 0.1	50.25^e^ ± 0.04
2	1320.23^c^ ± 19.36	65.35^d^ ± 0.02	66.15^d^ ± 0.32
4	1918.69^c^ ± 11.20	79.23^c^ ± 0.02	75.23^c^ ± 0.24
6	2623.56^b^ ± 9.15	82.15^b^ ± 0.02	86.51^b^ ± 0.30
Control	2915.55^a^ ± 13.5	100^a^	98.22^a^ ± 0.32
LSD = 0.05	71.22	0.81	0.94

Letters mean the noteworthy differences between treatments in line with Duncan’s check SE = Standard error.

#### Larval and pupal duration

According to the data as shown in Table 2, all of the checked chemicals considerably increased the larval duration, which was **8** (18.61 days), **3** (16.35 days), **2** (14.56 days), **6** (11.95 days), and **4** (14.65 days, respectively, according to control in (9.55 days). oppositely, the checked components reduced the pupal period with significantly different results from one another, tabulating as **8** (11.32 days) and **3** (13.56 days), while **2, 6,** and 4 had no significantly different results from one another, tabulating as 14.20, 15.33, and 16.85 days, respectively, compared to the larvae that were not treated (17.34 days).

#### Pupal weight

**Figure 3:**
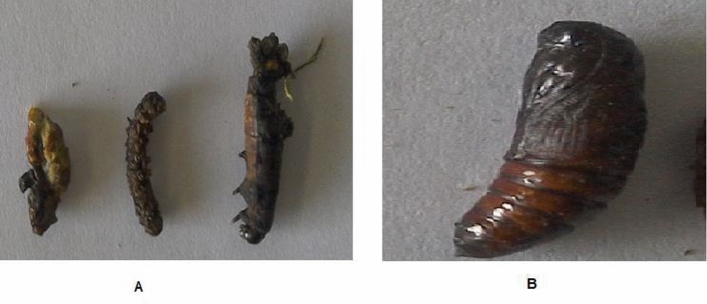
(**A**) Morphological malformations of *S. littoralis* (Boisd.) affected through checked products. (Pupae with larval head, larval legs and shortened malformed wings; non developed wings & remnants of larval prolegs). (**B**) Normal pupae (control).

According to result tabularized shown in Table 2, the pupal weight trended in the same direction. The checked components all significantly decreased pupal weight, with **8** being the most effective, recording (263.72 mg), followed by **3, 2, 6,** and **4** at 272.14, 281.60, 286.15, and 395.14, respectively, in comparison to the control pupal weight of 298.0 mg. As shown in Fig. 3, the malformations in *S. littoralis* caused by examined products. Pupae with a larval head, larval legs, and small, deformed wings; non-developed wings, and vestiges of larval prolegs. (B) Regular pupae (the control).

#### % of Normal, deformed pupae & adult emergency

The findings in Table 2, revealed that components were responsible for the latent effects **8, 3, 2, 6** and **4** were the most effective, recording (36.56, 72.51, 81.25, 90.25 and 94.68%), (13.23, 15.62, 7.65, 5.65 and 3.23%) and (73.35, 65.23, 68.31, 80.24 and 89.61%), respectively, associated to the control moiety (95.21, 3.20, 93.41%) to corresponding percentages of healthy pupae, malformed pupae, and adult emergence.

#### % of Fecundity and egg hatchability

Concerning to the results characterized of Table 3, the amount of eggs per female, the fecundity rate, and the hatchability rate, it was experiential that compounds **8** and **3** have been a markedly noteworthy diminution in the mean numbers of eggs laid via adult of females (fecundity), In the other hand, after treatment of the parent fourth instar larvae, eggs hatchability (fertility) was abruptly reduced in the offspring generation, with **8** recording 702.39 eggs per female, 22.8 fecundity, and 42.36% eggs hatchability, followed by 3 (956.33 eggs per female, 42.65 fecundity, and 50.25% eggs hatchability), in contrast to the control group (2915.55 eggs per female, 100 fecundity, and The least productive was number two (1320.23 eggs per female, 65.35 fecundity, and 66.15% fertility), while compound **6** exhibited (1918.69eggs/female, 79.23% fecundity & 75.23% fertility) & **4** exhibited (2623.56 eggs/female, 82.15 fecundity and 86.51% fertility).

## Structure-action relationship (SAR)

**Figure 4:**
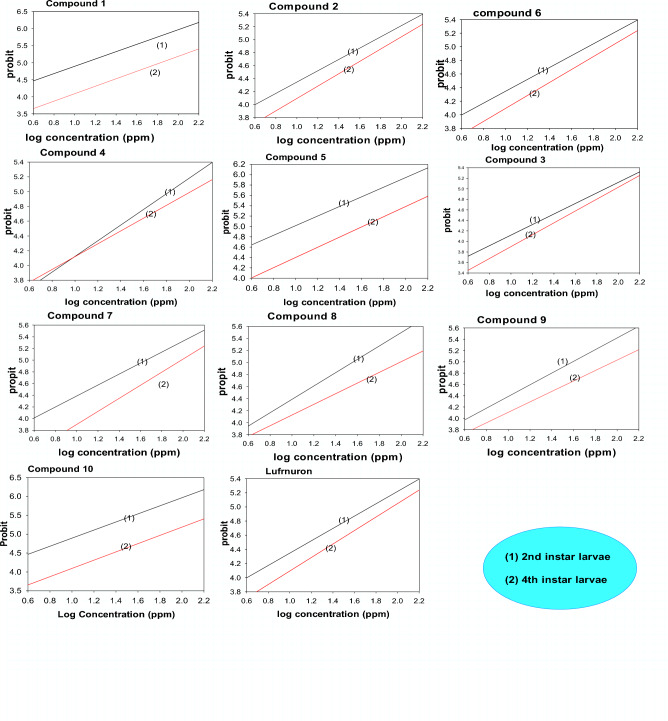
Insecticidal effectiveness of selective products **1–10** and lufenuron as reference insecticide for the 2nd & 4th larvae instar of *S. littoralis.*

**Figure 5:**
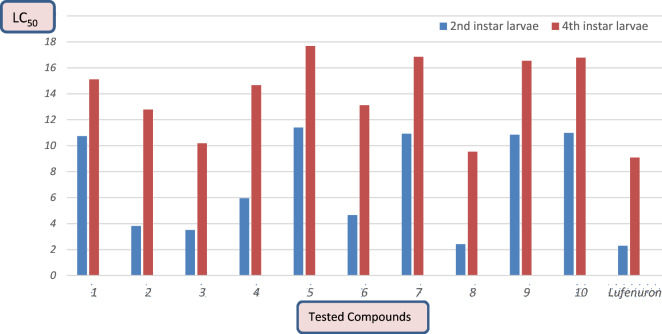
Insecticidal effectiveness of products **1–10** toward the 2nd & 4th larvae instar of *S. littoralis*.

Herein, the creation of novel, bioactive, polyfunctional substituted oxopropylthiourea compounds was deemed useful. By using a computerised regression analysis programme and confirming the toxicity value in Table 1 and Fig. 4, the median lethal concentration (LC_50_) & slope values of the targeted components were determined & expressed as parts per million (ppm). The insecticidal efficiency of the designed components (**1–10**) were compared with lufenuron teword *S. littoralis*, in which second instar larvae of *S. littarolis* are characterized via black lines & forth instar larvae of *S. littoralis* are signified via red lines after 3 days of treatment (Fig. 5). In this section the structure-action relationship was recognized component **8** is more performance toward 2nd & 4th larvae instar of *S. littoralis* insect than the other oxopropylthioureas. The high activity of product **8** this is occurrence of fluorophenyl, cyanoacetamide & carboxalic acid group in its structure. Existence of fluorophenyl & carbonitrile groups in this component which considered as an electron-withdrawing groups increase its effectiveness than the other oxopropylthioureas derivatives compared to the reference insecticide. Also, the product 3 gave high effective may be because of the existence of the fluorophenyl p-hydroxy phenyl, carboxalic acid group and piprazin moiety in its chemical structure. In addition to, the insecticidal activity of compound **2** showed higher toxicity might be because of the existence of fluorophenyl, p-methoxyphenyl, carboxalic acid group & piprazin group in its configuration. Finally, compound **8** is higher in toxicity than that of compound **3, 2, 4** & this because of the existence of fluorophenyl & cyanoacetamide in its building.

## Conclusion

In a continuation of our previous studies on searching a bout bioactive compounds, we reported here on the insecticidal activity of some oxopropylthiourea derivatives. The reaction mixture of 6-fluoro-7-[4-(isothiocyanatocarbonyl)piperazin-1-yl]-4-oxo-1,4-dihydroquinoline-3-carboxylic acid (**1**) in dry acetone, amine derivatives, conc. HCl and anhydrous ZnCl_2_ were allowed to reflux about 8 h. The crystal product was collection via filtration. On concerning the data that presented in Table 1 and illustrated in Figs. 4 and 5 which represented the insecticidal activity of novel oxopropylthiourea derivatives containing fluorophenyl and cyanoacetamide, we deduced that compound **8** is more efficacious toward second and forth *S. littoralis* larvae than the other oxopropylthioureas. The present data proved that values of LC_50_ of the most effected synthesized compound **8** was 2.412 ppm in which LC_50_ for commercial lufenuron was 2.295 ppm. On considering the toxicity line and slope we observed that the slope increase in the following order **8** > **3** > **2** > **6** > **4** > **1** > **9** > **7** > **10** > **5,** this order revealed that the homologous response of the treated strain of *S. littoralis* which presented variation in response against of target synthesized products. Additionally, in an effort to marginally enhance insecticidal compounds, evaluation of the latent effects of the examined components on several biological parameters, including adult longevity, pupal weight, proportion of normal, deformed pupae, & adult emergency, fecundity, & egg hatchability, was carried out. The high in effectiveness of component **8** may be because of the existence of fluorophenyl, cyanoacetamide and carboxalic acid group in their chemical structure. When compared to other oxopropylthioureas derivatives and the commercial lufenuron insecticide, the fluorophenyl and carbonitrile groups, which are thought of as electron-withdrawing groups, boost its efficiency.

## Experimental

### General method for preparing of oxopropylthiourea derivatives 2–10

A solution of target compound **1** (4 mmol) in dry acetone (20 mL), (4 mmol) of amine derivatives, (1 mL) conc. HCl and (0.2gm) anhydrous ZnCl_2_. For roughly 8 h, the reaction mixture was refluxed while being stirred. Ice-cold water was added once then the reaction had been cooled. After being filtered, the solid product was collected and dried.

#### 1-Ethyl-6-fluoro-7-(4-(((4-methoxyphenyl)carbamothioyl)glycyl)piperazin-1-yl)-4-oxo-1,4-dihydroquinoline-3-carboxylic acid 2

Pale yellow precipitate, Yield (60%). Mp 0.290–292 °C. FT IR (KBr) max cm^−1^: 3409 (OH, st), 3315, 3262 (NH), 3057 (CH-arom.) 2986–2835 (CH_2_, CH_3_, st), 1683 (C=O carboxylic), 1646 (C=O amide, st) and 1628 (C=C, st). ^1^H-NMR (ppm) 15.21 (s, 1H, COOH), 9.62(1H,NH), 8.95 (s, 1H, H-2 of quinolone); 8.38–7.93(m, 4H, arom.), 7.91(d, JH-F = 13 Hz, 1H, 5H of quinolone); 7.11 (d, JH-F = 7.5 Hz, 1H, 9H of quinolone); 6.11(1H, NH), 4.91 (s, 2H, CH_2_-CO), 4.60 (q, JH-H = 7 Hz, 2H, –CH_2_–CH_3_), 3.91 (s, 3H, OCH_3_), 3.76 (b, 2H, piperazine), 3.68 (b, 2H, piperazine), 3.41 (b, 4H, piperazine); 1.43 (t, JH-H = 7 Hz, 3H, –Me). ^13^CNMR (DMSO-d_6_), δ ppm: 176.62, 166.59, 149.17, 145.80, 144.01, 137.37, 107.03, 56.50, 49.90, 45.96, 19.14, 14.54. *Anal.* for C_26_H_28_FN_5_O_5_S (541.5) Calcd./found: C: 57.66/57.58, H: 5.21/5.19 and N: 12.93/12.91%.

#### 1-Ethyl-6-fluoro-7-(4-(((4-hydroxyphenyl)carbamothioyl)glycyl)piperazin-1-yl)-4-oxo-1,4-dihydroquinoline-3-carboxylic acid 3

Pale yellow precipitate, Yield (66%). Mp 0.298–300 °C. FT IR (KBr) max cm^−1^: 3408 (OH, st), 3314(OH), 3262, 3190 (NH), 3053 (CH-arom.) 2986–2835 (CH_2_, CH_3_, st), 1682 (C=O carboxylic), 1663 (C=O amide, st) and 1622 (C=C, st). ^1^H-NMR (ppm) 15.31 (s, 1H, COOH), 9.61 (s, 1H, NH), 9.60 (s, 1H, OH), 8.95 (s, 1H, 8H of quinolone); 8.38–7.93 (m, 4H, arom.), 7.91 (d, JH-F = 13 Hz, 1H, H-5 of quinolone); 7.21 (d, JH-F = 7.5 Hz, 1H, 8H of quinolone); 6.11(1H, NH), 4.91 (s, 2H , CH_2_–CO), 4.60 (q, JH-H = 7 Hz, 2H, –CH_2_–CH_3_), 3.76 (b, 2H, piperazine), 3.68 (b, 2H, piperazine), 3.41 (b, 4H, piperazine); 1.44 (t, JH-H = 7 Hz, 3H, -Me). ^13^CNMR (DMSO-d_6_), δ ppm: 177.19, 167.34, 166.94, 154.59, 152.16, 148.94, 145.63, 137.39, 119.98, 112.09, 107.61, 106.19, 56.79, 49.86, 45.63, 31.93, 19.50, 14.80. *Anal.* for C_25_H_26_FN_5_O_5_S (527.5) Calcd./found: C: 56.92/56.90, H: 4.97/4.95 and N: 13.27/13.25%.

#### 7-(4-((Benzylcarbamothioyl)glycyl)piperazin-1-yl)-1-ethyl-6-fluoro-4-oxo-1,4-dihydroquino-line-3-carboxylic acid 4

Pale yellow precipitate, Yield (60%). Mp 0.208–210 °C. FT IR (KBr) max cm^−1^: 3409 (OH, st), 3314, 3262(2NH), 3053 (CH-arom.) 2986–2835 (CH_2_, CH_3_, st), 1682 (C=O carboxylic), 1664 (C=O amide, st) and 1626 (C=C, st). ^1^H-NMR (ppm) 15.26 (s, 1H, COOH), 10.70 (s, 1H, NH), 8.94 (s, 1H, 2H of quinolone); 8.38–7.93(m, 4H, Harom.), 7.91(d, JH-F = 13 Hz, 1H, 5H of quinolone); 7.21 (d, JH-F = 7.5 Hz, 1H, 8H of quinolone); 6.11(1H,NH), 4.91(s, 2H, CH_2_-benz,),4.58 (s, 2H, CH_2_–), 4.60 (q, JH-H = 7 Hz, 2H, –CH_2_–CH_3_), 3.76 (b, 2H, piperazine), 3.68 (b, 2H, piperazine), 3.41 (b, 4H, piperazine); 1.44 (t, JH-H = 7 Hz, 3H, –Me). ^13^CNMR (DMSO-d_6_), δ ppm: 177.19, 167.34, 166.49, 154.59, 152.35, 145.94, 145.63, 137.39, 119.98, 112.09, 107.61, 106.19, 56.79, 50.01, 49.86, 45.63, 31.93, 19.50, 14.80. *Anal.* for C_25_H_26_FN_5_O_5_S (525.5) Calcd./found: C: 59.41/59.39, H: 5.73/5.70 and N: 13.32/13.29%.

#### 7-(4-((Cyclohexylcarbamothioyl)glycyl)piperazin-1-yl)-1-ethyl-6-fluoro-4-oxo-1,4-dihydroquino-line-3-carboxylic acid 5

Pale yellow precipitate, Yield (60%). Mp > 300 °C. FT IR (KBr) cm^−1^: 3408 (OH, st), 3315, 3264(2NH), 3053 (CH-arom.) 2986–2835 (CH_2_, CH_3_, st), 1681 (C=O carboxylic), 1663 (C=O amide, st) and 1626 (C=C, st). ^1^H-NMR (ppm) 15.26 (s, 1H, COOH), 8.94 (s, 1H, H-2 of quinolone); 8.38–7.93(m, 4H, arom.), 7.91(d, JH-F = 13 Hz, 1H, 5H of quinolone); 6.55 (s, 1H, NH) 7.21 (d, JH-F = 7.5 Hz, 1H, 8H of quinolone); 5.10 (s, 2H, CH_2_CO,), 4.58 (s,2H, CH_2_–), 4.60 (q, JH-H = 7 Hz, 2H, –CH_2_–CH_3_), 3.76 (b, 2H, piperazine), 3.82 (b, 2H, piperazine), 3.41 (b, 4H, piperazine); 1.80–1.27 (m,13H,cyclohexyl + -Me). ^13^CNMR (DMSO-d_6_), δ ppm: 177.29, 167.37, 167.05, 163.31, 145.94, 137.37, 119.52, 117.42, 112.32, 107.23, 56.27, 49.38, 45.41, 31.92, 23.91, 18.64, 14.77. *Anal.* for C_25_H_32_FN_5_O_4_S (517.6) Calcd./found: C: 58.01/58.00, H: 6.23/6.20 and N: 13.53/13.51%.

#### 1-Ethyl-6-fluoro-4-oxo-7-(4-((2-phenylhydrazine-1-carbonothioyl)glycyl)piperazin-1-yl)-1,4-dihydroquinoline-3-carboxylic acid 6

Pale brown precipitate, Yield (70%). Mp . 220–222 °C. FT IR (KBr) cm^−1^: 3409 (OH, st), 3314, 3261, 3190(3NH), 3053 (CH-arom.) 2986–2835 (CH_2_, CH_3_, st), 1683 (C=O carboxylic), 1663 (C=O amide, st) and 1627 (C=C, st). ^1^H-NMR (ppm) 15.26 (s, 1H, COOH), 10.20 (s,1H,NH), 8.89 (s, 1H, 2H of quinolone); 8.53–7.95 (m, 4H, H_arom_.), 7.91(d, JH-F = 13 Hz, 1H, H-5 of quinolone); 7.21 (d, JH-F = 7.5 Hz, 1H, H-8 of quinolone); 6.11(s, 1H, NH), 4.91(s, 2H, CH_2_CO,),4.58 (s,2H , CH_2_-), 4.60 (q, JH-H = 7 Hz, 2H, –CH_2_–CH_3_), 3.76 (b, 2H, piperazine), 3.82 (b, 2H, piperazine), 3.41 (b, 4H, piperazine); 1.44 (t, JH-H = 7 Hz, 3H, –Me). ^13^CNMR (DMSO-d_6_), δ ppm: 176.88, 166.89, 157.00, 149.29, 137.76, 126.37, 118.51, 111.94, 108.22, 106.77, 56.51, 49.88, 19.13, 14.69. *Anal.* for C_25_H_27_FN_6_O_4_S (526.5) Calcd./found: C: 57.02/57.00, H: 5.17/5.15 and N: 15.96/15.94%.

#### 1-Ethyl-6-fluoro-7-(4-((hydrazinecarbonothioyl)glycyl)piperazin-1-yl)-4-oxo-1,4-dihydroquino-line-3-carboxylic acid 7

Pale yellow precipitate, Yield (50%). Mp. 250–252 °C. FT IR (KBr) cm^−1^: 3408 (OH, st), 3314, 3263, 3190 (NH_2_, NH), 3053 (CH-arom.) 2986–2835 (CH_2_, CH_3_, st), 1681 (C=O carboxylic), 1664 (C=O amide, st) and 1627 (C=C, st). ^1^H-NMR (ppm) 15.21 (s, 1H, COOH), 8.95 (s, 1H, H-2 of quinolone); 8.00 (d, JH-F = 13 Hz, 1H, 5H of quinolone); 7.57 (d, JH-F = 7.5 Hz, 1H, H-8 of quinolone);7.27, 7.22(d, 2H, 2NH), 6.11(s, 2H, NH_2_), 4.62(s, 2H, CH_2_CO,),4.58 (s,2H, CH_2_–),3.76 (b, 2H, piperazine), 3.82 (b, 2H, piperazine), 3.41 (b, 4H, piperazine); 1.44 (t, JH-H = 7 Hz, 3H, -Me). ^13^CNMR (DMSO-d6), δ ppm: 176.34, 167.81, 166.18, 148.78, 137.42, 119.93, 112.02, 108.13, 106.40, 56.82, 47.64, 31.93, 18.58, 14.80. *Anal.* for C_19_H_23_FN_6_O_4_S (487.1) Calcd./found: C: 50.66/50.64, H: 5.15/5.12 and N: 18.66/18.64%.

#### 7-(4-((2-(2-Cyanoacetyl)hydrazine-1-carbonothioyl)glycyl)piperazin-1-yl)-1-ethyl-6-fluoro-4-oxo-1,4-dihydroquinoline-3-carboxylic acid 8

Yellow precipitate, Yielding compound (50%). Melting point. > 300 °C. FT IR (KBr) max cm^−1^: 3414 (OH, st), 3400–3190 (3NH), 3053 (CH-arom.) 2986–2835 (CH_2_, CH_3_, st), 2088(CN), 1708 (CO carboxylic), 1661 (CO amide, st) and 1628 (C=C, st). ^1^H-NMR (ppm) 15.21 (s, 1H, COOH), 9.13(s,1H,NH), 8.95 (s, 1H, H-2 of quinolone); 7.99(d, JH-F = 13 Hz, 1H, H-5 of quinolone); 7.69 (d, JH-F = 7.5 Hz, 1H, H-8 of quinolone);7.64(s, 2H, 2NH), 7.22(s 1H, 1NH), 4.62(s, 2H, CH_2_CO,), 4.16 (s, 2H, CH_2_-),3.75 (b, 2H, piperazine), 3.82 (b, 2H, piperazine), 3.41(b, 4H, piperazine); 2.89 (s,2H,CH_2_CN), 1.43 (t, JH-H = 7 Hz, 3H, -Me). ^13^CNMR (DMSO-d_6_), δ ppm: 178.62, 167.68, 166.46, 151.28, 149.48, 145.33, 137.73, 120.55, 111.91, 110.42, 106.24, 56.77, 49.57, 47.98, 42.90, 31.23, 19.10, 14.91. *Anal.* for C_22_H_24_FN_7_O_5_S (517.5) Calcd./found: C: 51.06/51.04, H: 4.67/4.65 and N: 18.95/18.92%.

#### 7-(4-(((2-Ethoxy-2-oxoethyl)carbamothioyl)glycyl)piperazin-1-yl)-1-ethyl-6-fluoro-4-oxo-1,4-dihydroquinoline-3-carboxylic acid 9

Yellow precipitate, Yield (53%). Mp > 300 °C. FT IR (KBr) cm^−1^: 3410 (OH, st), 3400–3190 (2NH), 3053 (CH-arom.) 2986–2835 (CH_2_, CH_3_, st), 1703 (C=O ester), 1690C=O, acid, st), (1662 (C=O amide, st) and 1628 (C=C, st ). ^1^H-NMR (ppm) 15.24 (s, 1H, COOH), 9.20(s,1H,NH), 8.95 (s, 1H, H-2 of quinolone); 8.43(d, JH-F = 13 Hz, 1H, 5H of quinolone); 7.96 (d, JH-F = 7.5 Hz, 1H, H-8 of quinolone); 7.28(s, 1H, NH), 5.09(q,2H, CH_2_-ester), 4.60–4.28(m, 4H, 2CH_2_,),4.25 (s,2H,CH_2_-),3.75 (b, 2H, piperazine), 3.82 (b, 2H, piperazine), 3.41(broad, 4H, piperazine); 1.95–187(t, 3H, CH_3_ ester), 1.43–1.35 (t, JH-H = 7 Hz, 3H, –CH_3_). ^13^CNMR (DMSO-d6), δ ppm: 177.14, 166.70, 154.32 152.56, 149.56, 139.35, 128.66, 117.57, 108.27, 106.56, 57.97, 49.26, 47.14, 42.88, 29.01, 25.72, 15.27. *Anal.* for C_23_H_28_FN_5_O_6_S (521.5) Calcd./found: C: 52.97/52.94, H: 5.41/5.40 and N: 13.43/13.40%.

#### 7-(4-(((2-Aminoethyl)carbamothioyl)glycyl)piperazin-1-yl)-1-ethyl-6-fluoro-4-oxo-1,4-dihydroquinoline-3-carboxylic acid 10

Pale yellow precipitate, Yield (60%). Mp 0.270 °C. FT IR (KBr) cm^−1^: 3421 (OH, st), 3057 (CH–arom.) 2986–2835 (CH_2_, CH_3_, st), 1693 (C=O carboxylic), 1646 (C=O amide, st) and 1628 (C=C, st). ^1^H-NMR (ppm) 15.20 (s, 1H, COOH), 9.45(s,1H,NH),8.95 (s, 1H, H-2 of quinolone); 7.97(d, JH-F = 13 Hz, 1H, 5H of quinolone); 7.90 (d, JH-F = 7.5 Hz, 1H, H-8 of quinolone); 4.63 (q, JH-H = 7 Hz, 2H, –CH_2_–CH_3_), 3.76 (b, 2H, piperazine), 3.67 (b, 2H, piperazine), 3.41 (b, 4H, piperazine); 2.74 (s,1H, NH), 1.43 (t, JH–H = 7 Hz, 3H, –CH_3_). ^13^CNMR (DMSO-d_6_), δ ppm: 176.68, 166.45, 151.28, 149.48, 145.33, 137.73, 120.95, 111.91, 107.74, 107.42, 107.34, 56.77, 42.90, 31.23, 19.10, 14.91. *Anal.* for C_40_H_46_F_2_N_10_O_8_S_2_ (896.9) Calcd./found: C: 53.56/53.54, H: 5.17/5.15 and N: 15.62/15.61% (Supplementary information 1) .

### Supplementary Information

Supplementary Figures.

## Data Availability

All data generated or analysed during this study are included in this published article (and its supplementary information files).

## References

[1] Oliveira C, Auad A, Mendes S, Frizzas M (2014). Crop losses and the economic impact of insect pests on Brazilian agriculture. Crop Prot..

[2] Yadav RS, Kumar D, Singh U, Singh D (2015). Insect-pests complex of cabbage in eastern Uttar Pradesh. Veg. Sci..

[3] Guo J, Qi J, He K, Wu J, Bai S, Zhang T, Zhao J, Wang Z (2019). The Asian corn borer Ostrinia furnacalis feeding increases the direct and indirect defence of mid-whorl stage commercial maize in the field. Plant Biotechnol. J..

[4] Abdelhamid AA, Elwassimy MM, Aref SA, Gad MA (2019). Chemical design and bioefficacy screening of new insect growth regulators as potential insecticidal agents against *Spodoptera littoralis* (Boisd.). Biotechnol. Rep..

[5] Abdelhamid AA, Salama KSM, Elsayed AM, Gad MA, El-Remaily MAAA (2022). Synthesis and toxicological effect of some new pyrrole derivatives as prospective insecticidal agents against the cotton leafworm, *spodoptera littoralis* (Boisduval). ACS Omega.

[6] Aydin H, Gürkan M (2006). The efficacy of spinosad on different strains of *Spodoptera littoralis* (Boisduval) (Lepidoptera: Noctuidae). Turk. J. Biol..

[7] Isman MB (2015). A renaissance for botanical insecticides?. Pest Manag. Sci..

[8] Isman MB, Miresmailli S, Machial C (2011). Commercial opportunities for pesticides based on plant essential oils in agriculture, industry and consumer products. Phytochem. Rev..

[9] Khamis WM, El-Desouky SE, Gad AA (2016). Toxicity and antifeedant effects of Apricot kernel extract and its main components against cotton leaf worm, *Spodoptera littoralis* (Lepidoptera: Noctuidae) larvae with reference to some physiological effects. Alex. Sci. Exch. J..

[10] Janzen D, Hallwachs W (2019). Perspective: Where might be many tropical insects?. Biol. Conserv..

[11] Wellinga K, Mulder R, Daalen VJJ (1973). Synthesis and laboratory evaluation of 1-(2,6-disubstituted benzoyl)-3-phenylureas, a new class of insecticides. II. Influence of the acyl moiety on insecticidal activity. J. Agric. Food Chem..

[12] Murray, A., Siddi. G., Vietto, M., Jacobson, R. M., & Thirugnanam, M. RH-7988: a new selective systemic aphicide. In Proc. of Brighton Crop Protection Conference-Pests and Diseases. **1**, 73–809(1988).

[13] Jadhav MR, Oulkar DP, Shabeer TPA, Banerjee K (2015). Quantitative screening of agrochemical residues in fruits and vegetables by buffered ethyl acetate extraction and LC-MS/MS analysis. J. Agric. Food Chem..

[14] Zhang LJ, Li Y, Wang K, Qin AF, Chen XG, Feng ZQ (2015). Synthesis and anti-proliferative activity evaluation of sorafenib derivatives with a 3-arylacryloyl hydrazide unit. Med. Chem. Res..

[15] Yang Y, Liu Y, Song H, Li Y, Wang Q (2016). Design, synthesis, insecticidal activity, and structure-activity relationship (SAR): Studies of novel triazone derivatives containing a urea bridge group based on transient receptor potential (TRP) channels. Mol. Divers..

[16] Abdelhamid AA, Elsaghier AMM, Aref SA, Gad MA, Ahmed NA, Abdel-Raheem SHAA (2021). Preparation and biological activity evaluation of some benzoylthiourea and benzoylurea compounds. Curr. Chem. Lett..

[17] Gad MA, Aref SA, Abdelhamid AA, Elwassimy MM, Abdel-Raheem ShAA (2021). Biologically active organic compounds as insect growth regulators (IGRs): Introduction, mode of action, and some synthetic methods. Curr. Chem. Lett..

[18] Abdelhamid AA, Aref SA, Ahmed NA, Elsaghier AMM, Abd El Latif FM, Al-Ghamdi SN, Gad MA (2023). Design, Synthesis, and Toxicological Activities of Novel Insect Growth Regulators as Insecticidal Agents against Spodoptera littoralis (Boisd). ACS Omega.

[19] Ali MA, Salah H, Gad MA, Youssef MAM, Elkanzi NAA (2022). Design, synthesis, and SAR studies of some novel chalcone derivatives for potential insecticidal bioefficacy screening on *Spodoptera frugiperda* (Lepidoptera: Noctuidae). ACS Omega.

[20] Hatem AE, Aldebis HK, Osuna EV (2011). Effects of the *Spodoptera littoralis* granulovirus on the development and reproduction of cotton leafworm *S. littoralis*. Biol. Control..

[21] Eppo PM (2015). *Spodoptera littoralis*, *Spodoptera litura**, **Spodoptera frugiperda Spodoptera eridania*. Eppo. Bull..

[22] El-Gaby MSA, Ammar YA, Drar AM, Gad MA (2022). Insecticidal bioefficacy screening of some chalcone and acetophenone hydrazone derivatives on *Spodopetra Frugiperda* (Lepidoptera: Noctuidae). Curr. Chem. Lett..

[23] Bakhite EA, Marae IS, Gad MA, Mohamed SK, Mague JT, Abuelhassan S (2022). Pyridine derivatives as insecticides. Part 3 synthesis, crystal structure, and toxicological evaluation of some new partially hydrogenated isoquinolines against *Aphis gossypii* (Glover). J. Agric. Food Chem..

[24] Abbott WS (1925). A method of computing the effectiveness of an insecticide. J. Econ. Entomol..

[25] Finny DJ (1952). Probit Analysis: A Statistical Treatment of the Sigmoid Response Curve.

[26] Sun YP (1950). Toxicity index—an improved method of comparing the relativetoxicity of insecticides. J. Econ. Entomol..

[27] Subbotina JO, Fabian WMF, Tarasov EV, Volkova NN, Bakulev VA (2005). Synthetic and theoretical aspects of new dimroth rearrangement of 6-aminopyran-2-ones to 6-hydroxypyridin-2-ones via carbamoyl ketenes. Eur. J. Org. Chem..

[28] Abu-Melha S (2018). Synthesis, modeling study and antioxidants activity of new heterocycles derived from 4-antipyrinyl-2-chloroacetamidothiazoles. Appl. Sci..

[29] Gad MA, Alqurashi EA, Alsenani NI, Abd El Latif FM, Aref SA, Ahmed NA, Abdelhamid AA, El-Saghier AMM (2003). Insecticidal activity, and SAR studies of semicarbazide, thiosemicarbazide, urea and thiourea derivatives against *Spodoptera littoralis* (Boisd). J. Umm Al-Qura Univ. Appll. Sci..

[30] Fadda AA, Abd El Salam M, Tawfik EH, Anwar EM, Etman HA (2017). Synthesis and insecticidal assessment of some innovative heterocycles 947 incorporating a thiadiazole moiety against the cotton leafworm *Spodoptera littoralis*. RSC Adv..

[31] Bhongade BA, Talath S, Gadad RA, Gadad AKJ (2016). Saudi Chem. Soc..

[32] Soliman N, Abd El Salam M, Abdel-Motaa M, Fadda AA (2020). Synthesis, Characterization and Biochemical impacts of some new bioactive 2 sulfonamide thiazole derivatives as potential insecticidal agents against the cotton 3 leafworm *Spodoptera littoralis*. J. Agric. Food Chem..

