# Supplementary material for: Synthesis and insecticide evaluation of some new oxopropylthiourea compounds as insect growth regulators against the cotton leafworm, Spodoptera littoralis
Source: Sci Rep. 2023 Aug 11;13:13089. doi: 10.1038/s41598-023-39868-y (PMC10421850; doi:10.1038/s41598-023-39868-y)
Supplement: Supplementary file 1 — Supplementary Figures. [file 41598_2023_39868_MOESM1_ESM.docx]

**Synthesis, Insecticide Evaluation & SAR Studies of Some New Oxopropylthiourea compounds Linked to Norfloxacin as Insect Growth Regulators against the leafworm, *Spodoptera littoralis* (Boisd.)**

**Ahmed M. El-Saghier*^1^, Laila Abosella^1,2^, Gamal A. Aborahma^3^, Esmail O. Elakesh^4^, Antar A. Abdelhamid^1,5^, Mohamed A. Gad^6^**

^1^ Chemistry Department, Faculty of Science, Sohag University, 282524, Sohag, Egypt.

^2^ Medicinal Chemistry Department, Faculty of Pharmacy, **Sabratha University, Sabratha**, Libya.

^3^ Medicinal Chemistry Department, Faculty of Pharmacy, Minia University, Minia, Egypt.

^4^ Chemistry Department, Faculty of Science, **Al Zawiya University, Al Zawiya**, Libya.

^5^ Chemistry Department, Faculty of Science, Albaha University, Albaha 1988, Saudi Arabia

^6^ Research Institute of Plant Protection, Agriculture Research Center, 12112 Giza, Egypt.

.


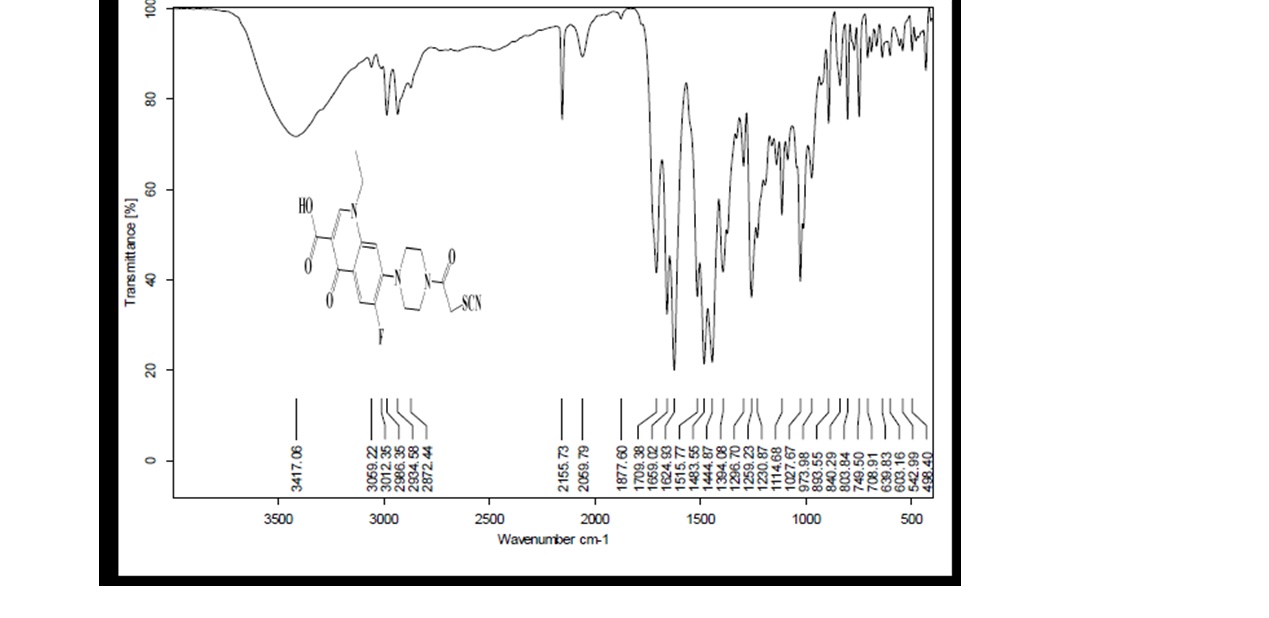


**Figure 1**: IR Spectrum of compound **1**


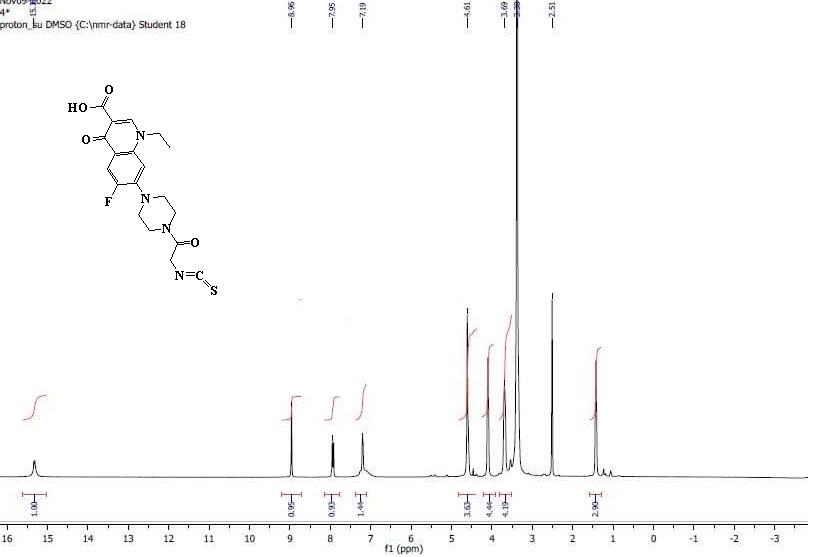


**Figure 2**: ^1^HNMR Spectrum of compound **1**


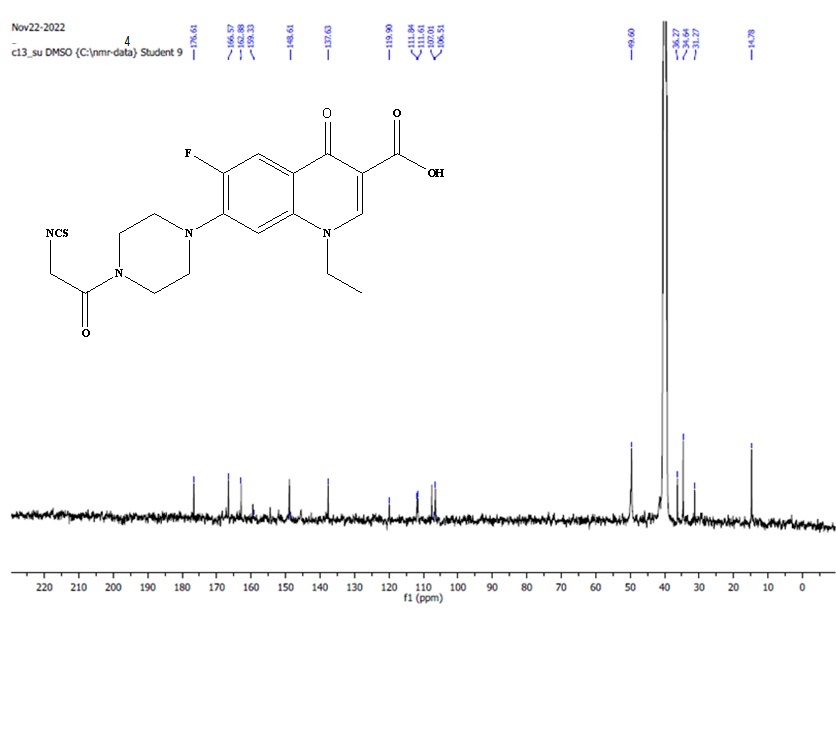


**Figure 3**: ^13^CNMR Spectrum of compound **1**


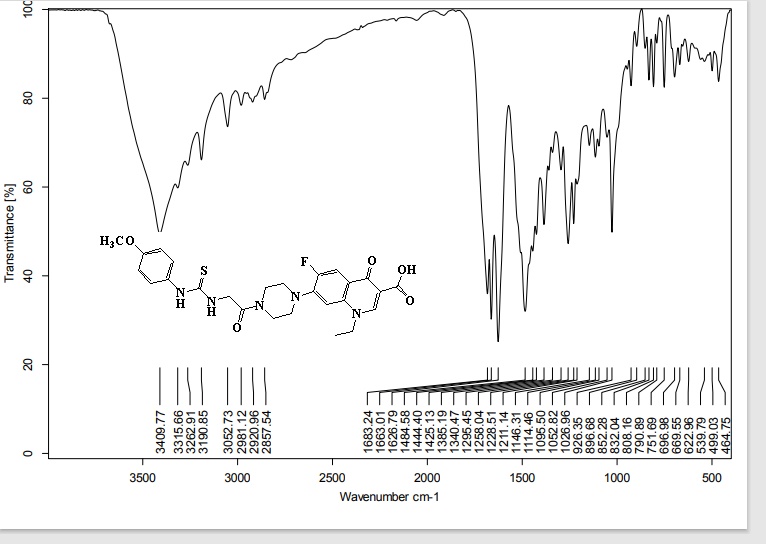


**Figure 4**: IR Spectrum of compound **2**


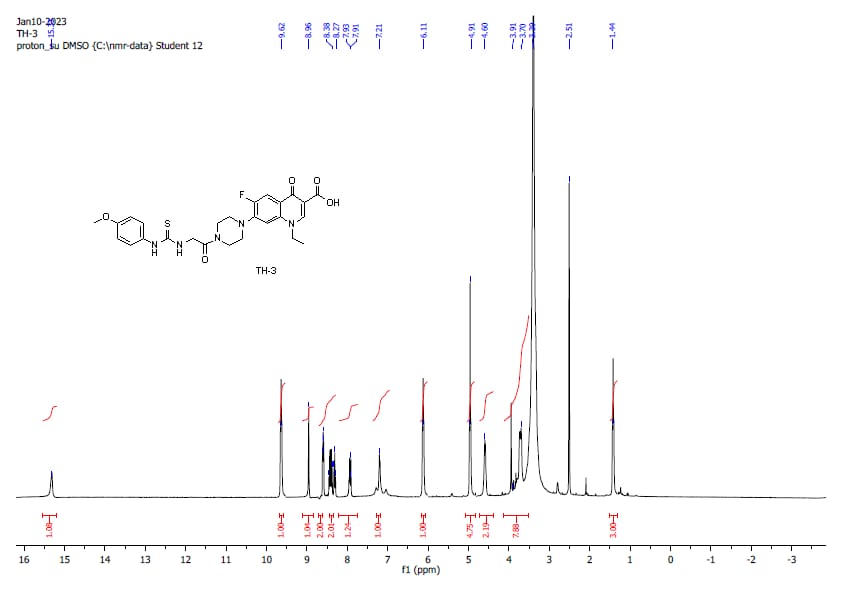


**Figure 5**: ^1^HNMR Spectrum of compound **2**


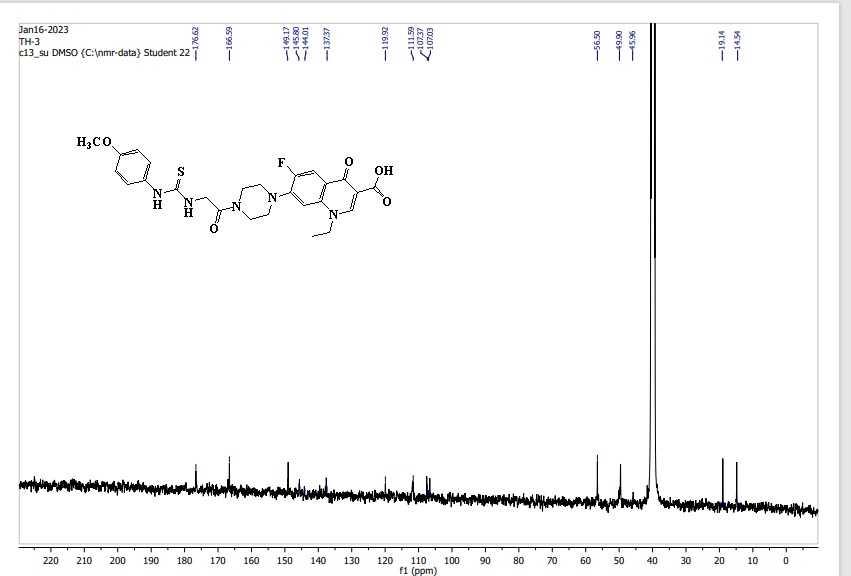


**Figure 6**: ^13^CNMR Spectrum of compound **2**


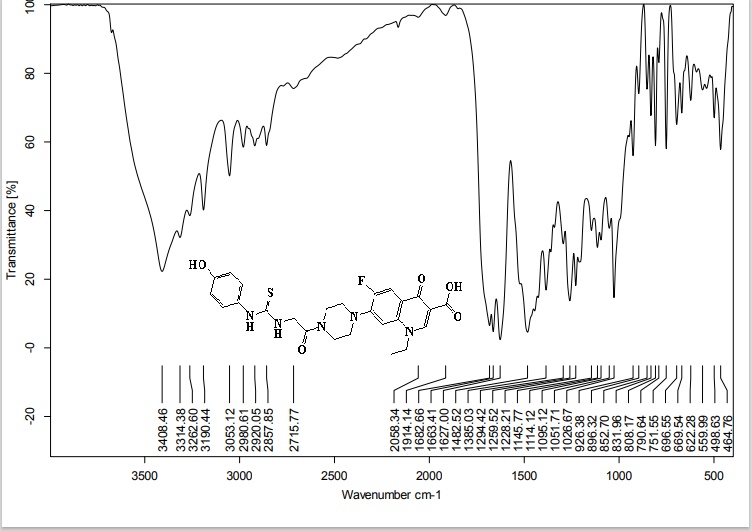


**Figure 7**: IR Spectrum of compound **3**


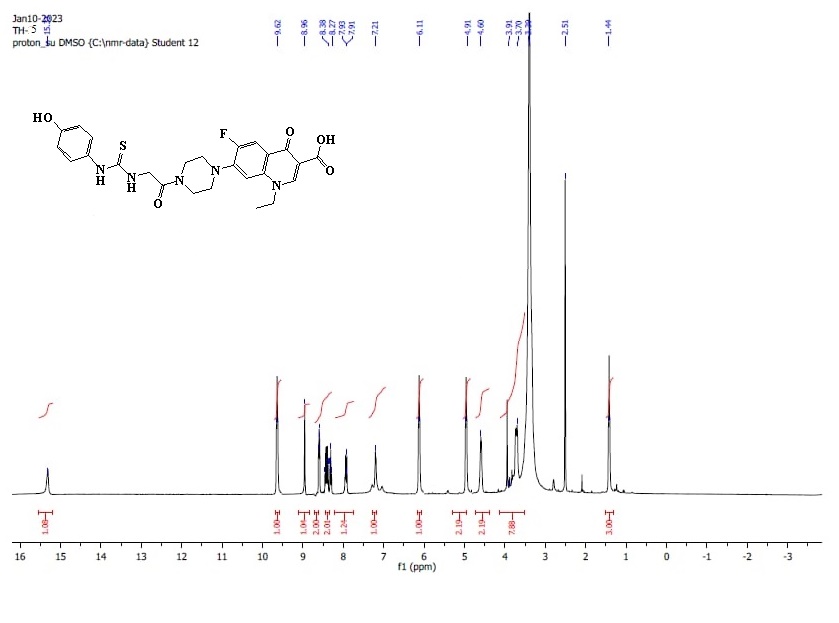


**Figure 8**: ^1^HNMR Spectrum of compound **3**


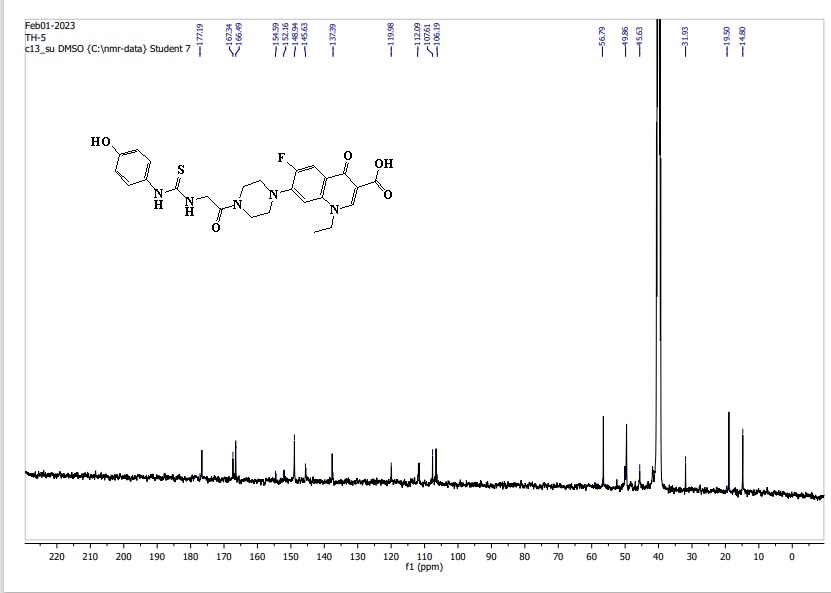


**Figure 9**: ^13^CNMR Spectrum of compound **3**


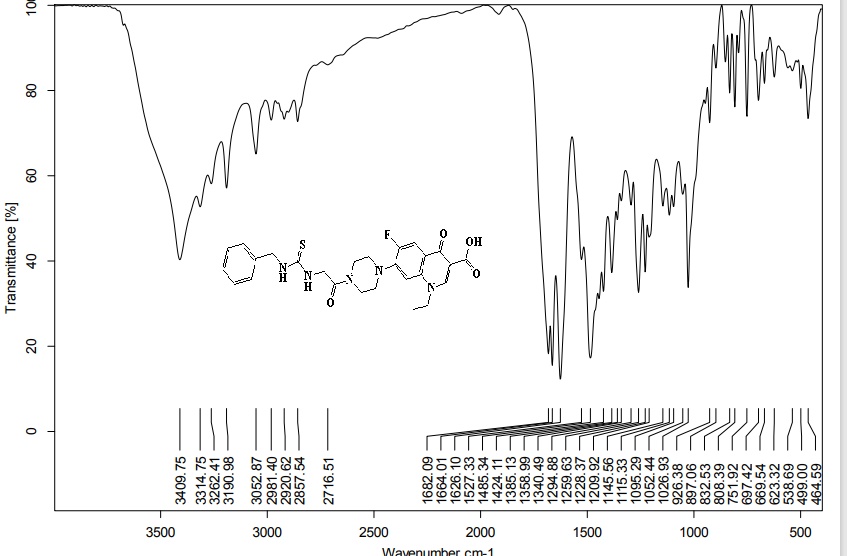


**Figure 10**: IR Spectrum of compound **4**


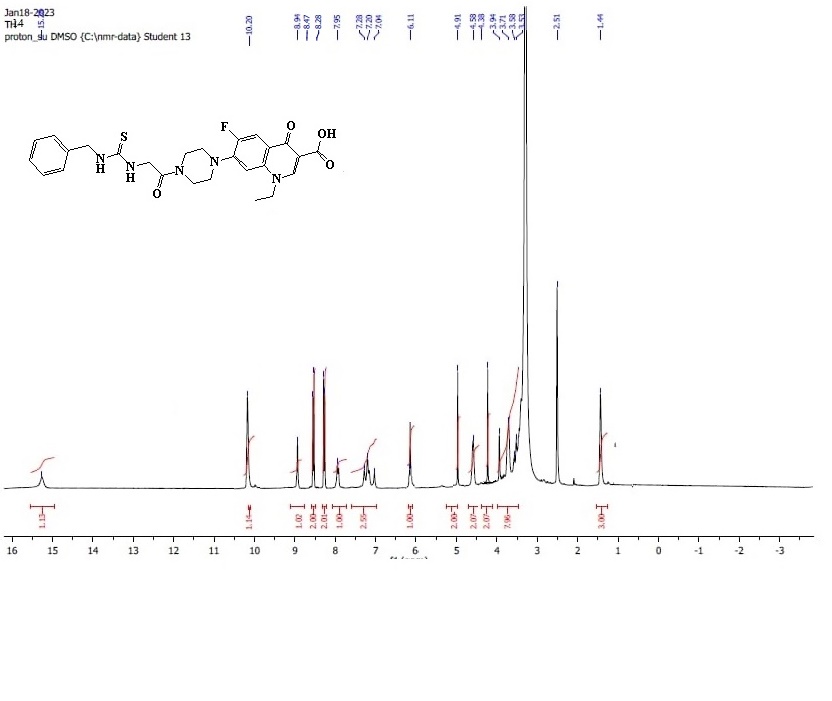


**Figure 11**: ^1^HNMR Spectrum of compound **4**


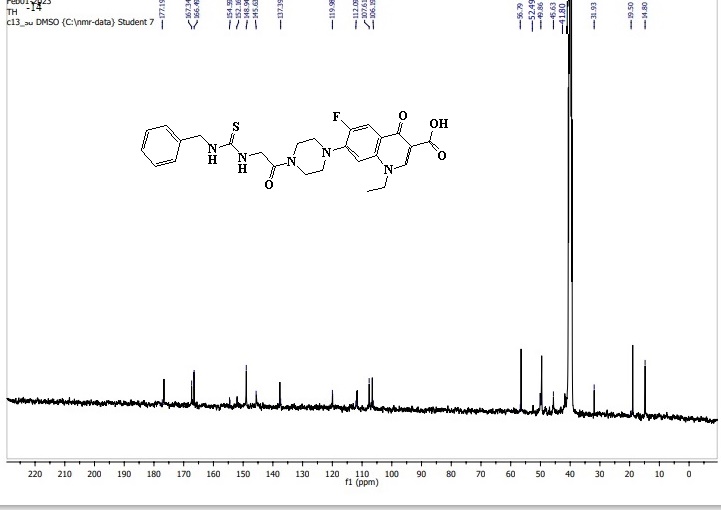


**Figure 12**: ^13^CNMR Spectrum of compound **4**


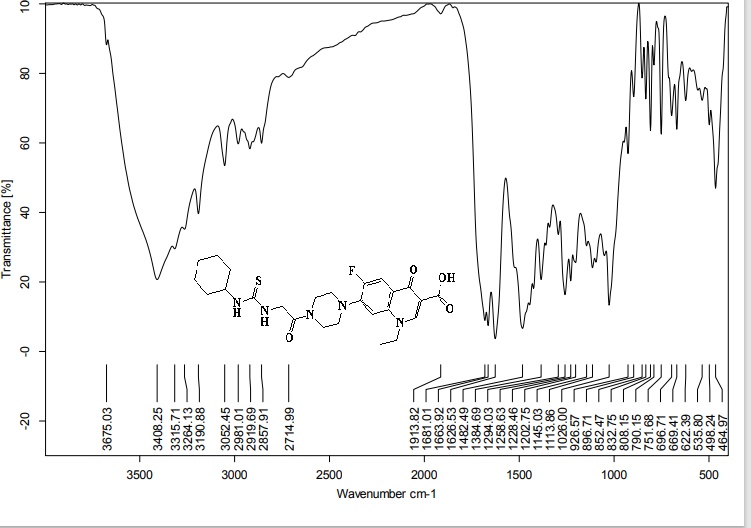


**Figure 13**: IR Spectrum of compound **5**


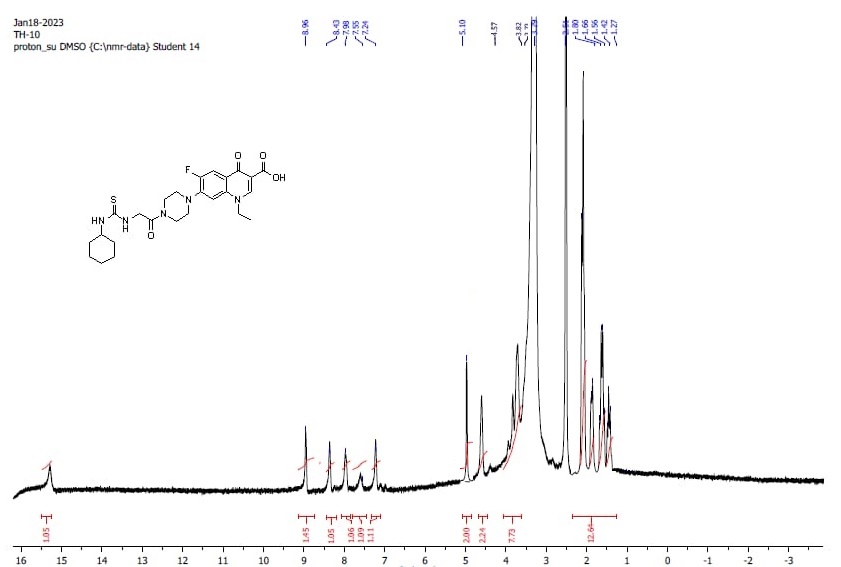


**Figure 14**: ^1^HNMR Spectrum of compound **5**


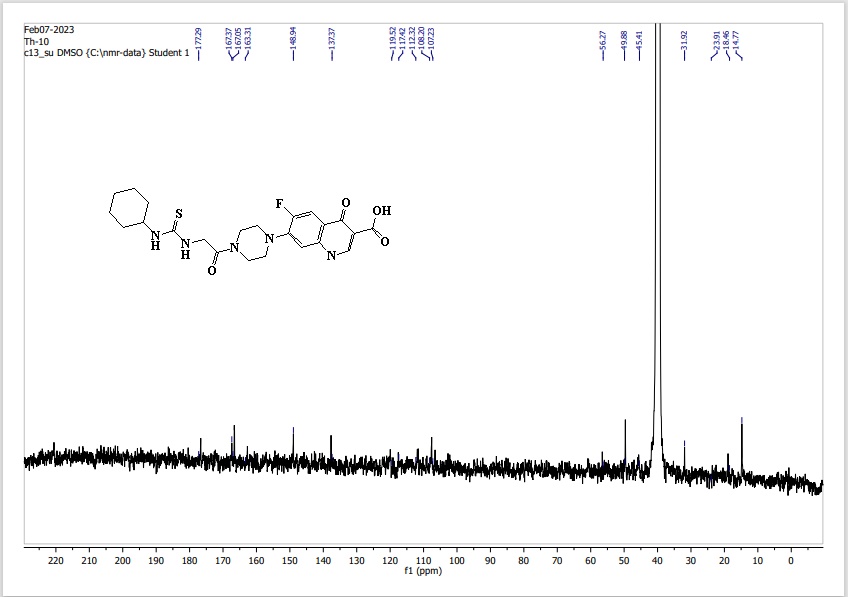


**Figure 15**: ^13^CNMR Spectrum of compound **5**


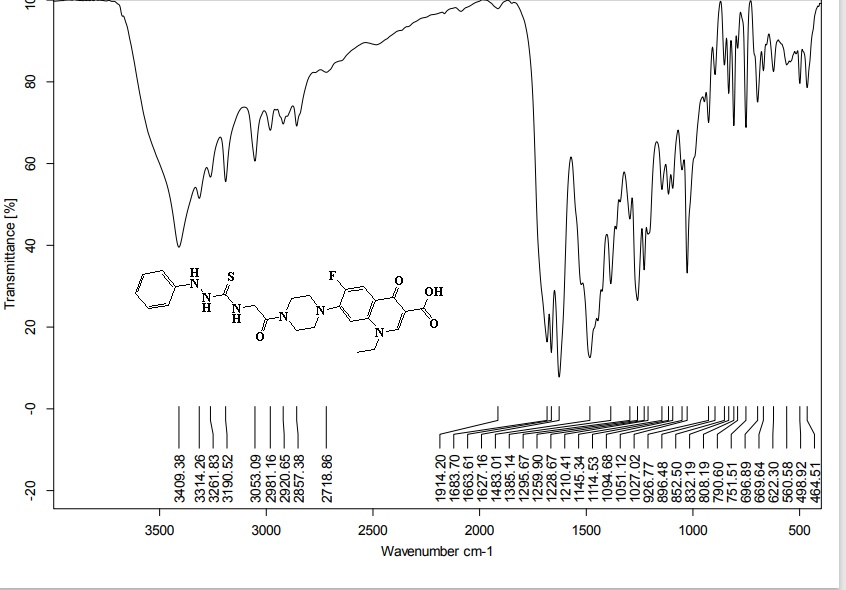


**Figure 16**: IR Spectrum of compound **6**


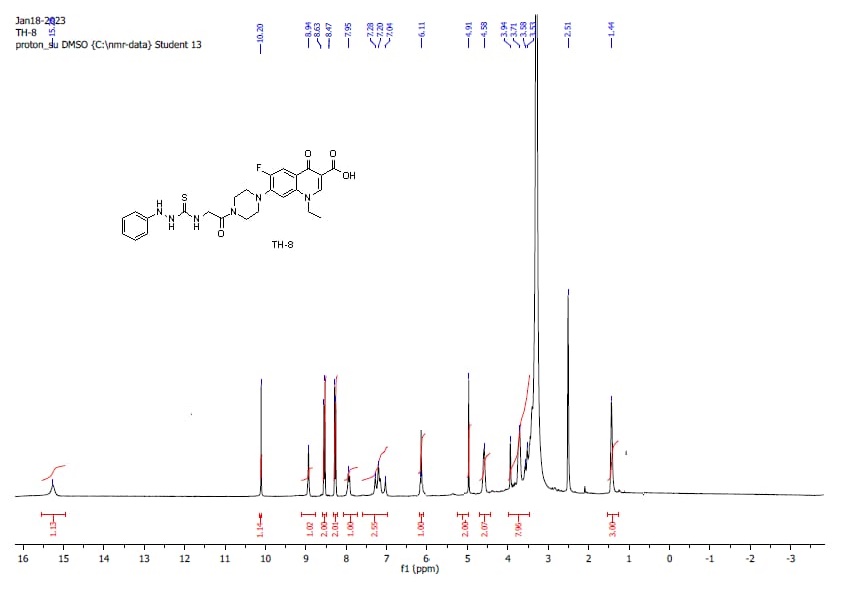


**Figure 17**: ^1^HNMR Spectrum of compound **6**


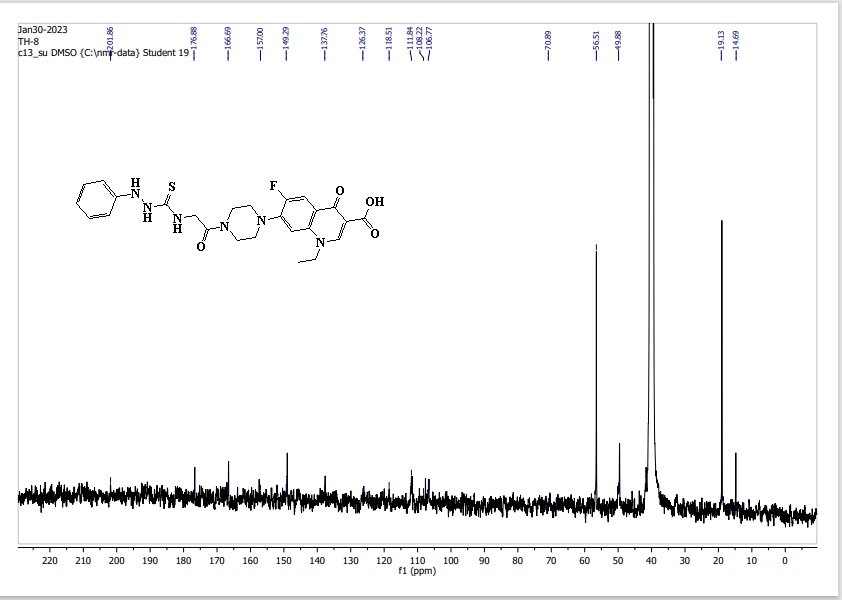


**Figure 18**: ^13^CNMR Spectrum of compound **6**


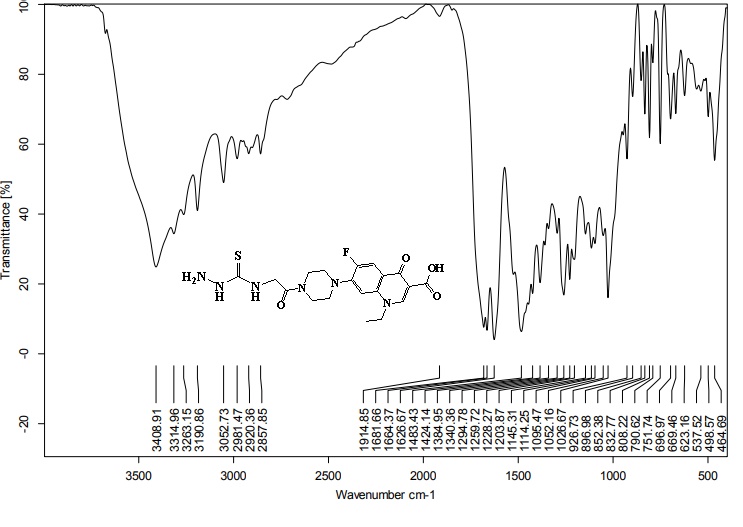


**Figure 19**: IR Spectrum of compound **7**


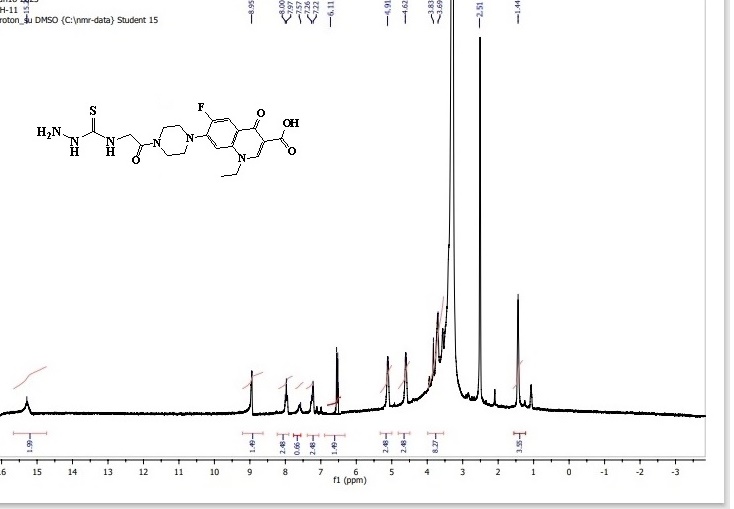


**Figure 20**: ^1^HNMR Spectrum of compound **7**


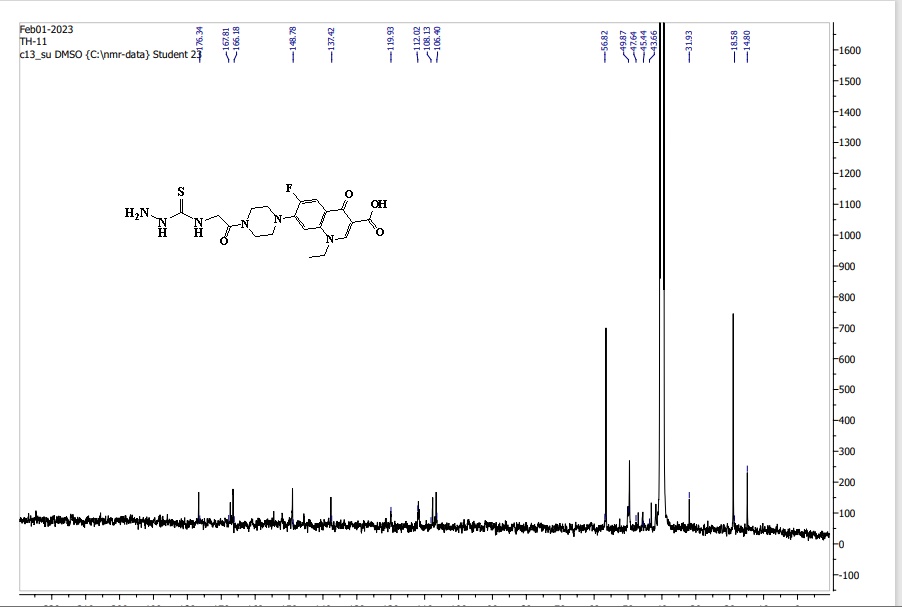


**Figure 21**: ^13^CNMR Spectrum of compound **7**


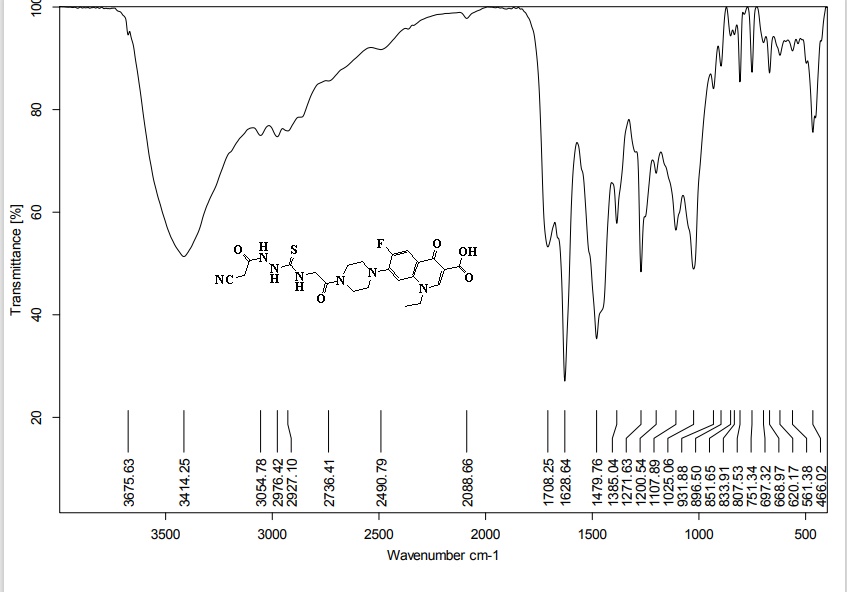


**Figure 22**: IR Spectrum of compound **8**


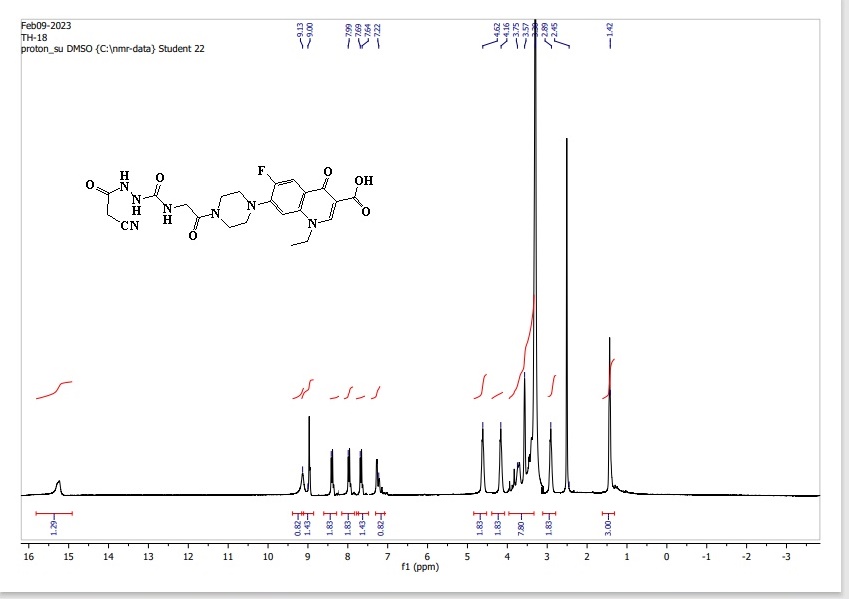


**Figure 23**: ^1^HNMR Spectrum of compound **8**


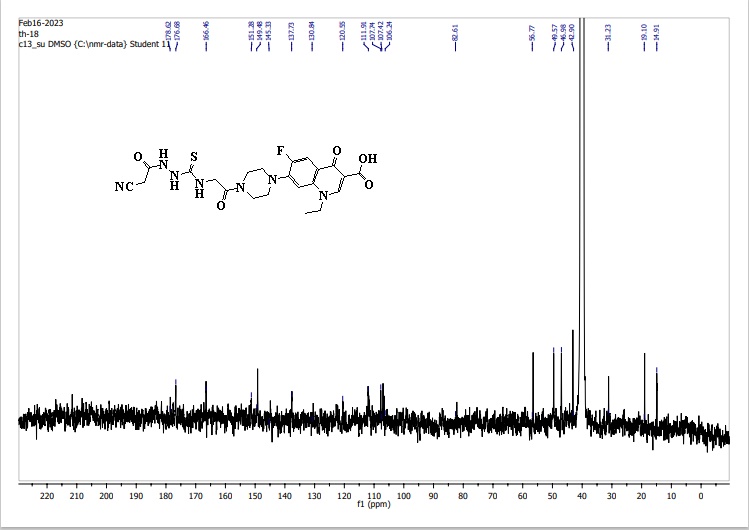


**Figure 24**: ^13^CNMR Spectrum of compound **8**


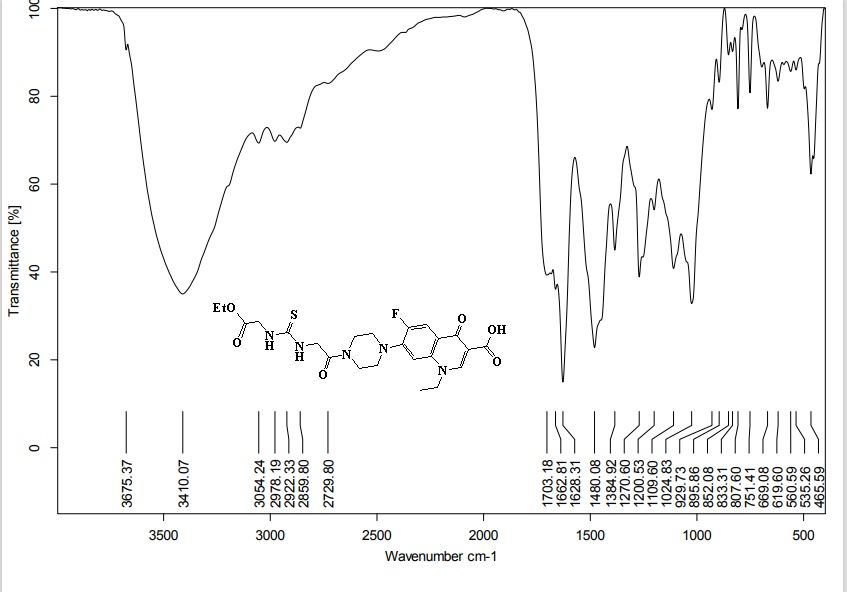


**Figure 25**: IR Spectrum of compound **9**


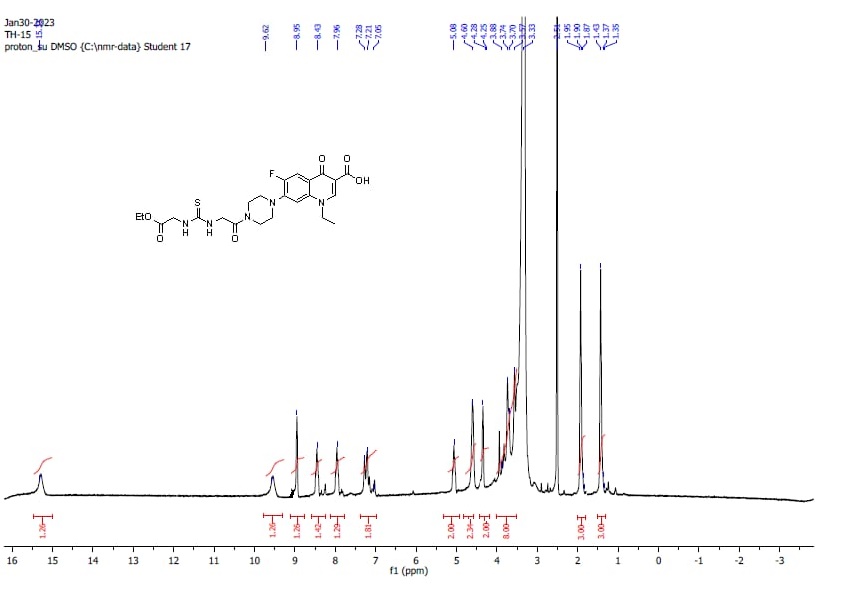


**Figure 26**: ^1^HNMR Spectrum of compound **9**


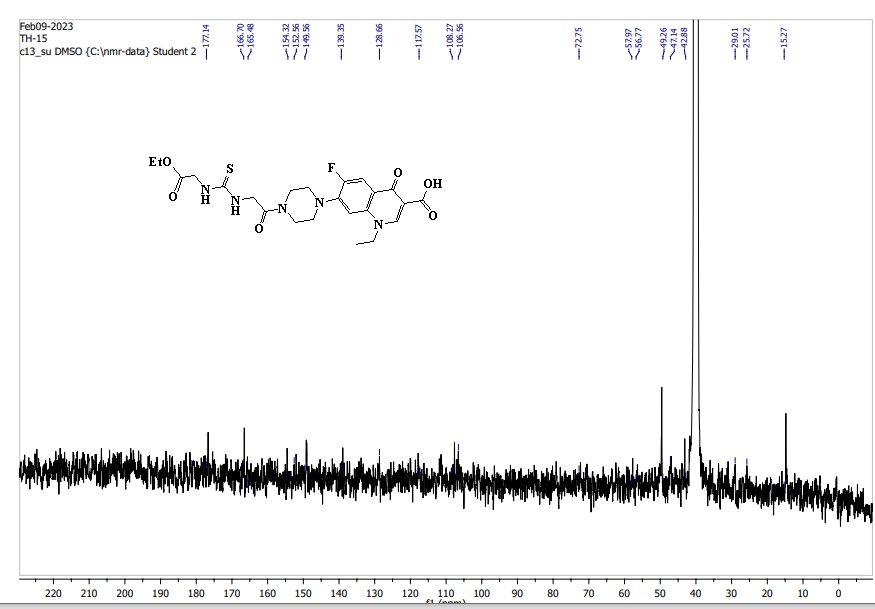


**Figure 27**: ^13^CNMR Spectrum of compound **9**


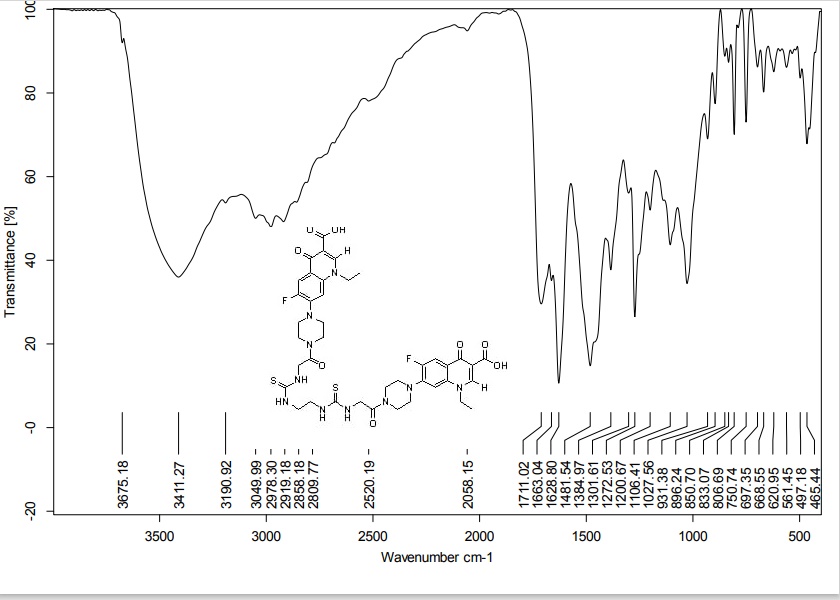


**Figure 28**: IR Spectrum of compound **10**


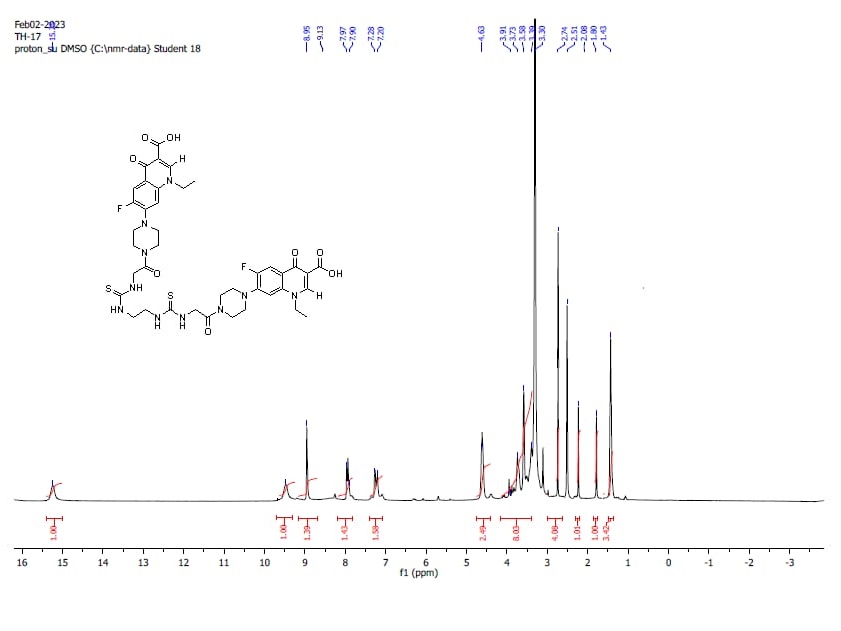


**Figure 29**: ^1^HNMR Spectrum of compound **10**


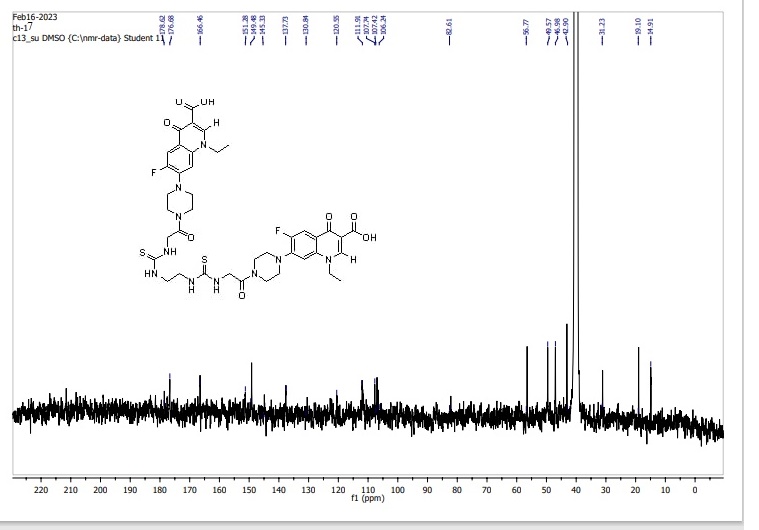


**Figure 30**: ^13^CNMR Spectrum of compound **10**
